# Design of a real-time energy monitoring system for profiling consumption in IoT nodes during local data processing

**DOI:** 10.1016/j.ohx.2026.e00764

**Published:** 2026-03-23

**Authors:** Alvaro A. Villa-Garzón, Jhon W. Branch-Bedoya, Fernan A. Villa-Garzón

**Affiliations:** Facultad de Minas, Universidad Nacional de Colombia, Carrera 80 No 65-223, Medellín, Colombia

**Keywords:** Real-time power monitoring, IoT energy profiling, Energy-performance trade-off, IoT power monitoring, Green IoT

## Abstract

This work presents the development of a two-part platform designed to evaluate the energy performance of Internet of Things (IoT) devices. The first component is a real-time monitoring system capable of synchronously measuring voltage and current to estimate the energy consumption of IoT nodes during the execution of computationally intensive algorithms. The second component is a battery-powered IoT test node that includes a user interface for configuring the percentage of training data used in a linear regression algorithm (RedWine dataset). Experimental evaluation shows that the monitoring system provides stable and accurate electrical measurements, while the test node enables controlled variation of computational load. Results demonstrate a nearly linear relationship between energy consumption, processing time, and the proportion of training data, with evidence that reduced datasets can achieve comparable model accuracy at significantly lower energy cost. The proposed platform provides a practical tool for analyzing the trade-off between algorithmic performance and resource usage, supporting the design of more energy-efficient IoT systems

## Graphical abstract

**Figure:**
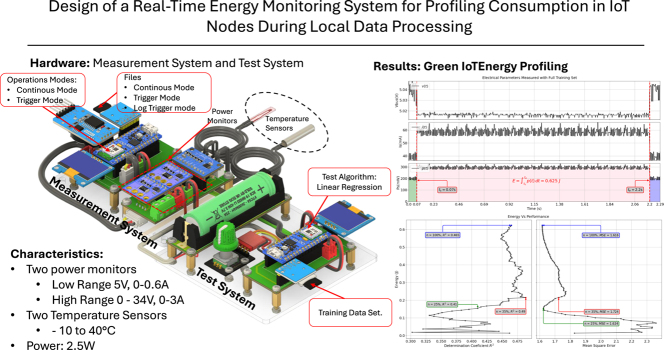

## Specifications table

**Table:** 

Hardware name	Electronic system for energy consumption profiling and monitoring of IoT devices, and test system

Subject area	Educational tools and open source alternatives to existing infrastructure

Hardware type	Electrical engineering and computer science

Closest commercial analog	• RD UM25C • FNIRSI FNB58

Open source license	CERN-OHL-S v2

Cost of hardware	$ 65 USD

Source file repository	Mendeley Data: 10.17632/hxdhnmv4mr.3

## 1 Hardware in context

The rapid expansion of connected Internet of Things (IoT) systems is reveled by Industry analyses estimating that more than 18 billion IoT devices were deployed worldwide in 2024, with projections exceeding 39 billion devices by 2030 [1]. As noted by Andriulo et al. (2024) [2], the limitations of centralized cloud computing regarding latency and privacy have driven a shift toward decentralized architectures, such as edge and hybrid computing. Consequently, as devices transition from simple sensing nodes to complex platforms executing on-device machine learning (ML), this has generated a growing demand for experimental tools capable of accurately characterizing the energy consumption of embedded devices. This need is particularly critical in battery-powered and energy-constrained applications, where power consumption directly determines system lifetime and operational sustainability. Energy-aware design has therefore become a central topic in IoT research, especially within the context of edge computing and green IoT systems, where efficient resource utilization is essential for long-term autonomous operation [3].

Accurate energy profiling enables researchers to understand the relationship between software execution, hardware activity, and power consumption. Such knowledge is fundamental for optimizing embedded algorithms, designing energy-efficient firmware, and correctly dimensioning energy harvesting and storage subsystems in IoT deployments [3]. As IoT nodes increasingly incorporate local processing capabilities, including machine learning algorithms at the edge, the ability to measure and analyze energy consumption during specific computational tasks becomes even more important.

Recent methodological advances emphasize the importance of *task-specific energy profiling* as a decision-support tool for system-level design and microcontroller selection. Shinde et al. propose a framework that decomposes node operation into canonical workloads such as sensing, data processing, memory access, wireless communication, and sleep modes, and evaluates metrics including energy per task and energy per CPU cycle [4]. Their work demonstrates how high-resolution measurements can guide design trade-offs such as on-node processing versus raw data transmission, batching strategies, and microcontroller selection for energy-autonomous wireless sensor nodes. These results highlight the importance of measurement platforms capable of capturing synchronized electrical traces that can later be mapped to application-level workloads.

Existing instrumentation for energy profiling typically falls into two main categories: laboratory-grade power analyzers and low-cost commercial power meters. High-end laboratory instruments provide excellent measurement accuracy, high sampling rates, and advanced triggering capabilities, allowing precise correlation between energy consumption and software execution events. However, such instruments are typically expensive, bulky, and dependent on laboratory infrastructure, which limits their accessibility and practical use in many research environments.

At the opposite end of the spectrum, low-cost commercial USB power meters are widely available and easy to use. Devices such as the RD UM25C or FNIRSI FNB58 provide basic voltage and current measurements suitable for quick diagnostics [5], [6]. Nevertheless, these instruments are not designed for scientific experimentation. They generally lack autonomous data logging, precise timestamping mechanisms, and hardware synchronization capabilities with the device under test (DUT), which significantly limits their usefulness for detailed energy profiling experiments.

At the high end, precision DC power analyzers such as the Keysight N6705C represent the gold standard for embedded power profiling. As demonstrated by Wang et al. [7], these instruments provide high sampling rates and hardware-triggered synchronization required to benchmark neural networks on microcontrollers. However, their high cost ($15,000 USD), bulky form factor, and dependence on mains power make them impractical for widespread deployment in field experiments.

Several academic platforms have been proposed to bridge the gap between high-end laboratory equipment and simple commercial meters. One notable example is EMPIOT [8], an open-source energy measurement platform designed for wireless IoT devices. EMPIOT integrates measurement circuitry with a Raspberry Pi-based architecture, enabling programmable data acquisition and software-driven experimentation. While this approach provides flexibility and improved measurement capabilities compared to basic USB meters, it relies on a single-board computer running a full operating system. This dependency increases system complexity, power overhead, and cost, and limits its suitability for fully embedded or field-deployable measurement scenarios. In addition, EMPIOT relies on the INA219 monitoring integrated circuit, which provides 12-bit resolution for current measurements, limiting the achievable dynamic range when profiling modern ultra-low-power IoT devices.

Another significant contribution in this area is the eProfiler system [9], which introduces a high-precision power monitoring platform capable of measuring currents ranging from the nanoampere to the milliampere scale. The system supports GPIO-based state tracing, enabling correlation between device states and power consumption events, and reports an average measurement error of approximately 0.45%. Despite its high measurement accuracy, the system primarily focuses on electrical energy measurements and does not explicitly incorporate environmental variables that may influence power consumption during long-term experiments.

Addressing the challenge of high dynamic range (HDR) power measurement in IoT loads is an active research area. Recent approaches, such as the switched-capacitor instrumentation proposed by Tehrani and Atarodi [10], achieve extremely wide dynamic ranges but rely on complex custom circuitry and offer limited software integration for application-level profiling.

Beyond instrumentation platforms, recent research on Green IoT systems has highlighted the importance of accurate energy characterization for the design of sustainable sensing infrastructures. Kuaban et al. [3] present an analytical and experimental framework for optimizing energy harvesting and storage subsystems in IoT deployments for pipeline monitoring. Their study demonstrates that reliable estimation of node energy consumption is essential for correctly dimensioning photovoltaic panels, batteries, and duty-cycling strategies. In such scenarios, experimental energy profiling becomes a key step for validating theoretical models and ensuring long-term operational autonomy of sensor networks.

Similar solutions for monitoring electrical variables have also been proposed in the literature. One example is the system known as Jericho [11], which was designed as a meteorological monitoring station that additionally measures electrical variables such as current and voltage in photovoltaic generation systems. Although this platform provides integrated environmental and electrical monitoring capabilities, its current sensing stage relies on a Hall-effect sensor. While Hall-effect sensors offer electrical isolation and ease of integration, they typically exhibit slower response times and may be susceptible to external magnetic field interference. These limitations can reduce measurement accuracy and temporal resolution when profiling fast current variations in embedded electronic systems.

Despite these advances, an intermediate solution remains necessary: a compact, low-cost, and open-source energy monitoring platform capable of performing high-resolution measurements while operating independently from laboratory equipment or host computers. Furthermore, most existing solutions focus exclusively on electrical parameters and rarely consider the influence of environmental conditions on measured energy consumption. In CMOS-based embedded systems, temperature variations can significantly affect leakage currents and static power dissipation, potentially introducing measurement bias during long experimental runs.

To address these limitations, this work proposes a standalone energy profiling platform specifically designed for embedded IoT experimentation. The system integrates high-resolution voltage and current monitoring with autonomous data logging, hardware synchronization triggers, and thermal sensing capabilities. The platform is built using widely available components and follows an open-source hardware approach, facilitating reproducibility and adoption in academic research environments.

An additional capability of the proposed platform is its dual-channel measurement architecture. The system integrates two independent energy monitoring channels, allowing simultaneous acquisition of voltage and current from two distinct subsystems of an IoT node. This capability enables comparative energy analysis between different components of the system, such as the microcontroller and the wireless communication module, or between sensing and processing subsystems. Dual-channel profiling is particularly useful in edge computing scenarios where energy trade-offs between local processing and wireless transmission must be evaluated experimentally.

In addition to electrical measurements, the proposed system incorporates a thermal profiling subsystem that enables the simultaneous acquisition of ambient and device temperature. This capability allows researchers to correlate variations in energy consumption with temperature fluctuations, providing deeper insight into the behavior of embedded systems during long-duration experiments.

Table 1 presents a comparison between representative academic energy measurement platforms and the system proposed in this work.

The proposed system aims to provide a practical experimental platform for investigating the energy-performance trade-offs of embedded algorithms executed on IoT nodes. By enabling precise, synchronized, temperature-aware, and multi-channel energy measurements, the platform supports the development of energy-efficient embedded applications and facilitates reproducible experimentation in IoT and edge computing research.

**Table 1:** Comparison of representative IoT energy monitoring platforms.

System	Type	Resolution	Dynamic range	Meas. channels	Standalone operation	Hardware Sync.	Thermal profiling
EMPIOT [8]	SBC-based platform	12-bit	Moderate	1	No	No	No

eProfiler [9]	Dedicated energy meter	High precision	Very high (nA–mA)	1	Partial	Yes	No

Green IoT Testbed [3]	IoT characterization framework	Experimental profiling	Application-dependent	1	No	No	No

Task-specific profiling [4]	Profiling methodology	Instrument-dependent	Task-level metrics	1	No	Yes	No

Tehrani [10]	Analog addition	16-bit	High (nA–A)	**1**	No	Yes	No

Proposed system	Embedded monitoring platform	16-bit	High (μA–A)	**2**	Yes	Yes	Yes

## 2 Hardware description

**Fig. 1:**
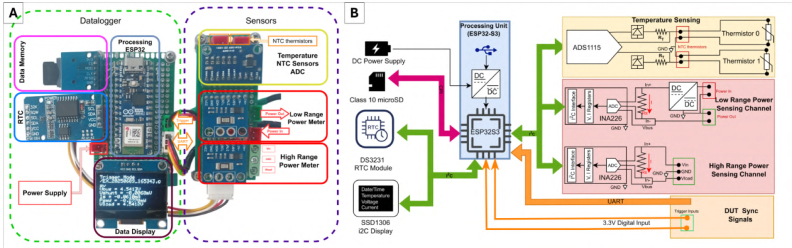
Overview of the proposed energy profiling platform, (A) Assembled prototype, (B) Hardware block diagram.

The proposed system is a standalone, high-resolution energy monitoring instrument designed specifically for profiling embedded software. Unlike generic USB power meters, the architecture prioritizes autonomous operation, thermal correlation, and hardware-level synchronization. The platform employs a dual-channel sensing stage controlled by an Espressif ESP32-WROOM-32 microcontroller. The system block diagram is presented in Fig. 1.

### 2.1 Measurement front-end (Dual-channel architecture)

To address the challenge of high dynamic range (HDR) in IoT devices — capturing both sleep currents in the microampere range and transmission bursts in the ampere range — the system utilizes a dual-channel high-side sensing architecture based on the Texas Instruments INA226. This 16-bit monitoring IC provides significantly higher resolution (2.5μV LSB) compared to the 12-bit INA219 used in previous academic prototypes [8].

The design incorporates two specialized measurement paths:

**High-Current Path (Direct Pass-Through):** This path is intended for high-power loads such as motors and cellular communication modules. It employs a 10mΩ shunt resistor ($R_{\mathrm{shunt1}}$) and supports input voltages up to 36 V. The source is directly connected to the load without voltage regulation.

**Low-Current Path (Regulated Source):** This path is optimized for profiling low-power microcontroller platforms. It employs a precision 0.1Ω shunt resistor ($R_{\mathrm{shunt2}}$) to enhance measurement resolution. The channel integrates a high-efficiency DC–DC buck converter (DFRobot DFR0569), which accepts an input voltage range of 3.0–15.0 V and provides a regulated 5.0 V output to the Device Under Test (DUT). This configuration allows the instrument to operate simultaneously as a power supply and an energy profiler.

### 2.2 Thermal profiling subsystem

Unlike conventional power meters, the proposed system accounts for the temperature dependence of static power consumption in CMOS-based IoT devices. The hardware integrates a thermal profiling stage based on two 10kΩ NTC thermistors (B=3950).

Temperature measurements are acquired using an external 16-bit ADC (ADS1115) rather than the internal ADC of the ESP32, which exhibits limited effective resolution and nonlinearity effects [12]. To further improve the measurement stability, a 2.5 V precision voltage reference (LM285D-2.5) is used for the thermistor sensing circuitry. This configuration ensures consistent and high-resolution thermal data suitable for long-term profiling experiments.

**Sensor configuration:** One thermistor measures ambient temperature ($T_{\mathrm{amb}}$), while the second is embedded in a compact probe designed to be coupled to the DUT package, providing case temperature ($T_{\mathrm{case}}$).

**Relevance:** This dual-sensing capability enables each energy measurement to be associated with thermal metadata, allowing separation of algorithmic energy costs from temperature-induced variations.

### 2.3 Processing and synchronization

The system is driven by an Arduino Nano ESP32-S3. This specific module was chosen over generic ESP32 development boards for three critical reasons: (1) Its industrial-grade PCB design minimizes electromagnetic interference (EMI) affecting the sensors; (2) The specific ESP32-S3 variant provides extended memory availability for future firmware updates involving edge-ML processing; and (3) The standardized form factor ensures long-term hardware availability for reproducibility.

This ESP32-S3 has a two core SoC, One core manages file operations, while the second executes the real-time data acquisition loop to ensure deterministic timing.

**Hardware synchronization triggers:** A dedicated GPIO interface allows the DUT to signal the start and end of specific software sections using a 3.3 V logic pulse. Upon detecting a rising edge, the system inserts a timestamped marker with millisecond latency.

**Timekeeping:** A DS3231 precision Real-Time Clock (RTC) provides absolute timestamps with ±2ppm accuracy, enabling reliable long-term field measurements.

### 2.4 Operational modes

The firmware supports two primary acquisition modes:

**Continuous monitoring mode:** Designed for long-term autonomous operation such as battery lifetime estimation. Voltage, current, and power samples are aggregated into one-minute statistical summaries (mean, minimum, and maximum) to reduce storage usage.

**Triggered profiling mode:** Intended for software-level energy auditing. The mode is activated by the hardware trigger and records raw measurements at approximately 500 Hz (2 ms sampling period). Data capture continues while the trigger signal remains asserted by the DUT.

### 2.5 Storage and visualization

The platform operates autonomously without the need for an external host:

**Autonomous logging:** Measurement data are stored locally on a microSD card in CSV format.

**Status display:** A 0.96-inch OLED display (128 × 64 px) provides real-time feedback including voltage, current, and active acquisition mode.

In summary, the proposed energy profiling platform provides a versatile tool for researchers across various domains. Its utility extends beyond basic power measurement, offering specific benefits for several laboratory tasks:

• **Optimization of Edge-ML algorithms:** The platform facilitates identifying the optimal trade-off between model accuracy and energy consumption, directly supporting the design and validation of energy-efficient “Green IoT” applications.
• **Simultaneous characterization of independent subsystems:** Through its dual-channel architecture, researchers can isolate and compare the electrical consumption of specific components, such as microcontrollers versus wireless communication modules, within a single test run.
• **Analysis of thermal-power dependencies:** The integration of dual temperature sensors allows for correlating ambient and case temperature variations with leakage currents in CMOS-based devices, which is critical for long-term reliability studies in variable environments.
• **Autonomous field experimentation:** The system’s ability to operate independently from a host computer — utilizing local microSD storage and a real-time OLED display — makes it ideal for performance testing in remote or real-world IoT deployments.
• **Automated hardware benchmarking:** By utilizing hardware synchronization triggers, the platform can autonomously capture high-resolution power profiles synchronized with specific software execution states, significantly accelerating the benchmarking process for new embedded devices.

## 3 Design files summary

The complete design resources for the proposed energy monitoring system are available in the Mendeley Data repository (DOI: **10.17632/hxdhnmv4mr.3**). To facilitate reproducibility and ease of navigation, the repository structure has been consolidated into three primary directories:

• **Hardware Design (/Hardware):** Contains the source CAD files (Autodesk Fusion 360 archive), standard Gerber/NC Drill files for PCB manufacturing, and the Bill of Materials (BOM).
• **Firmware Source Code (/Firmware):** Contains the C++ firmware for the ESP32 microcontroller, organized as a PlatformIO/Arduino project. External dependencies (e.g., *Adafruit_INA226*, *RTClib*) and compilation instructions are documented in the repository’s configuration files.
• **Validation Hardware (/Validation_Node):** As a supplementary resource, the design files for the “Load Emulation Node” used for validation (refer to Appendix) are provided in a separate subdirectory.

In accordance with open-science best practices, all hardware and software files are released under the **CERN Open Hardware Licence Version 2 - Strongly Reciprocal (CERN-OHL-S v2)** to ensure transparency and derivative freedom (see Table 2).

**Table 2:** Summary of design files available in the repository.

Design filename/folder	File type	Open source license	Location
/Hardware/PCB_Production	Gerbers, Drill, Pick&Place	CERN-OHL-S v2	Repository
/Hardware/Source_CAD	Fusion 360 Archive (.f3z)	CERN-OHL-S v2	Repository
/Firmware/ESP32_Meter	Arduino Source Code	CERN-OHL-S v2	Repository
/Validation_Node (Appendix)	CAD & Firmware	CERN-OHL-S v2	Repository
/BOM	Excel BOM	CERN-OHL-S v2	Repository
/Results and config file	Config.txt and csv	CERN-OHL-S v2	Repository

## 4 Bill of materials summary

In the Mendeley Data repository 10.17632/hxdhnmv4mr.3, a directory named BOM contains a detailed list of the components used to assemble both systems, including itemized descriptions and direct purchase links for each component.

The total cost for replicating the proposed measurement system is approximately **$65.00 USD**. While basic data loggers can be constructed for less, this design prioritizes **measurement linearity and long-term stability** over minimum cost. Key architectural decisions include the use of an external 16-bit ADC (ADS1115) and a dedicated voltage reference to mitigate the noise and non-linearity typical of low-cost internal microcontroller ADCs.

Table 3 details the components required for the high-precision measurement instrument.

**Table 3:** Bill of materials for the precision measurement system.

Component	Description	Qty	Unit cost	Total (USD)
MCU	Arduino Nano ESP32-S3 (Official)	1	$19.30	$19.30
Primary sensor	INA226 Power Monitor (16-bit)	2	$3.37	$6.74
Thermal ADC	ADS1115 16-bit ADC Module	1	$3.21	$3.21
V-Ref	LM285 2.5 V Precision Reference	1	$1.23	$1.23
Power Reg	DFRobot 5 V Buck/Boost (DFR0569)	1	$2.90	$2.90
Display	1.3${}^{\prime \prime }$ OLED SH1106 (128 × 64)	1	$3.60	$3.60
RTC	DS3231 Precision RTC	1	$3.30	$3.30
Storage	MicroSD SPI Module + 16 GB Card	1	$5.14	$5.14
Sensors	NTC 10k Thermistors (B = 3950)	2	$2.00	$4.00
Connectors	JST-XH/2.54 mm Headers	Set	–	$4.50
Passives	Resistors (Metal Film) & Diodes	Set	–	$1.00
PCB	PCB Manufacturing (Prorated)	1	$10.00	$10.00

*Note*: Costs reflect retail pricing for single units. The Arduino Nano ESP32 was selected for industrial reliability; cost can be reduced by $15 using generic clones, though with potential stability trade-offs.

## 5 Build instructions

The assembly process is designed to be modular. First, the printed circuit boards (PCBs) are populated, followed by the hardware configuration of the sensing modules, and finally, the system integration. Comprehensive 3D assembly views and step-by-step wiring guides are available in the repository (/Hardware/Assembly).

### 5.1 PCB population

**Fig. 2:**
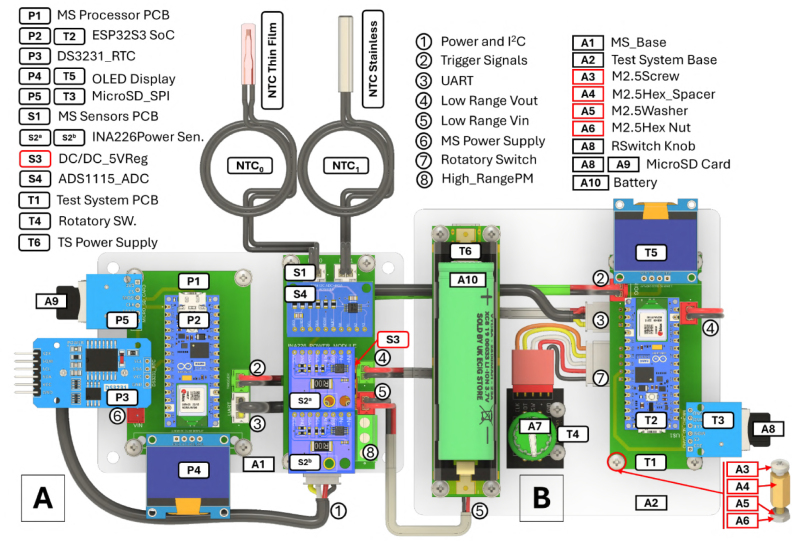
Complete system assembly diagram. The Measurement System (A) is shown connected to the validation hardware (B). Numbered tags correspond to the cable IDs listed in Table 4.

The Measurement System is split into two stacked PCBs to isolate the digital processing unit from the analog sensing front-end (see Fig. 2).

1. **Processor PCB:** Solder the female pin headers (2.54 mm pitch) corresponding to the ESP32 microcontroller, DS3231 RTC, and MicroSD module footprints. Install the OLED display using a 4-pin header. *Note: Ensure the ESP32 antenna area faces outward to minimize RF interference.*
2. **Sensors PCB:** Solder the screw terminals (5.08 mm pitch) for power inputs and outputs. Install the female headers for the INA226 modules, DC-DC converter, and ADS1115. Solder the passive components ($R_{ref},R_{NTC}$) directly onto the PCB footprints.

### 5.2 Sensor configuration (INA226)

**Fig. 3:**
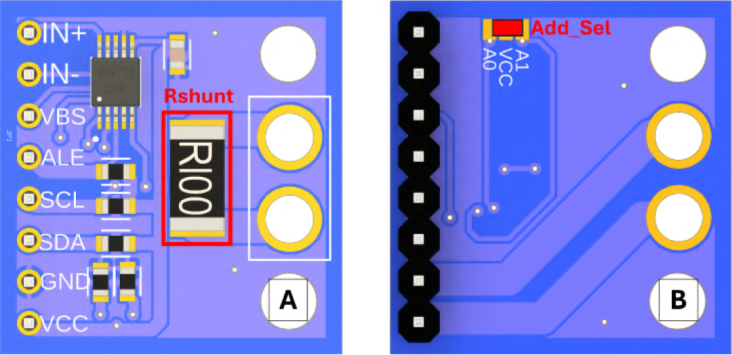
Configuration of the INA226 modules. (A) Top view shunt resistance replacement. (B) Bottom view High-Range configuration requiring the address bridge (A0).

Before installing the INA226 modules onto the Sensors PCB, they must be modified to define their $I^{2}C$ addresses and current ranges as shown in Fig. 3.

• **Low-Range Module (Address 0x40):** Leave address pads A0 and A1 open. Verify the default 0.1Ω shunt resistor is intact.
• **High-Range Module (Address 0x41):** Bridge the **A0** pad to $V_{CC}$ (or connect the *Add Sel* pin high). Desolder the default shunt and replace it with the external 0.01Ω precision shunt during final wiring.

### 5.3 System integration and wiring

Connect the Processor PCB and Sensors PCB using the dedicated $I^{2}C$/Power umbilical cable. Table 4 details the interconnection of external peripherals.

**Table 4:** System interconnection guide.

ID	Source connector	Destination	Signal description
1	MS_I2C_Pro (Proc. PCB)	MS_I2C_Sense (Sens. PCB)	Power (5 V/GND) + $I^{2}C$ Data Bus
2	MS_TRIG_IN	DUT/Test Node	Digital Trigger Input (3.3 V Logic)
3	MS_UART	External Logger/PC	Serial Data (TX/RX) for Debugging
4	NTC_0 /NTC_1	Thermistor Probes	Temperature inputs (Non-polarized)
5	High-range terminals	Load/Source	High current path (Direct Pass-Through)

## 6 Operation instructions

The operation of the measurement platform is designed to be autonomous. The following steps detail the procedure for preparing, configuring, and operating the system in its two primary modes: Continuous (Logger) and Triggered (Burst).

### 6.1 Safety and operating limits

Before connecting any Device Under Test (DUT), users must adhere to the following operational constraints. Exceeding these limits may result in permanent hardware damage.

1. **Logic Level Compliance (3.3 V Only):** The ESP32 microcontroller operates at **3.3 V logic**. All digital interfaces, including the **UART port** and **Trigger signals**, are **NOT 5 V tolerant**.
  • *Warning:* Connecting 5 V logic sources directly to the Trigger or UART pins will damage the SoC. Use a bidirectional logic level shifter if interfacing with 5 V equipment.
2. **Low-Range Path Limits (15 V/600 mA):** The Low-Range measurement path powers the DUT via a DC-DC Buck Converter (DFRobot DFR0569). This component imposes two critical limits:
  • **Input Voltage:** Maximum **15 V**. Exceeding this will destroy the regulator.
  • **Output Current:** Maximum **600 mA**. This path is strictly intended for low-power microcontrollers. Do not attempt to power Single Board Computers (SBCs) through this path.
3. **High-Range Current Limits (Thermal Constraints):** Although the shunt resistors are rated for higher currents, the **PCB trace width** limits the safe continuous operation.
  • **Continuous Current:** Up to **3 A**.
  • **Burst Current:** Up to **8 A** is permissible for short durations (<10 s). Prolonged operation at >3A without active cooling may cause PCB overheating.
4. **Common Ground:** The system shares a common ground reference between the USB power supply, measurement inputs, and the DUT. Do not connect to mains-referenced equipment without galvanic isolation.

### 6.2 Setup and connections

**Fig. 4:**
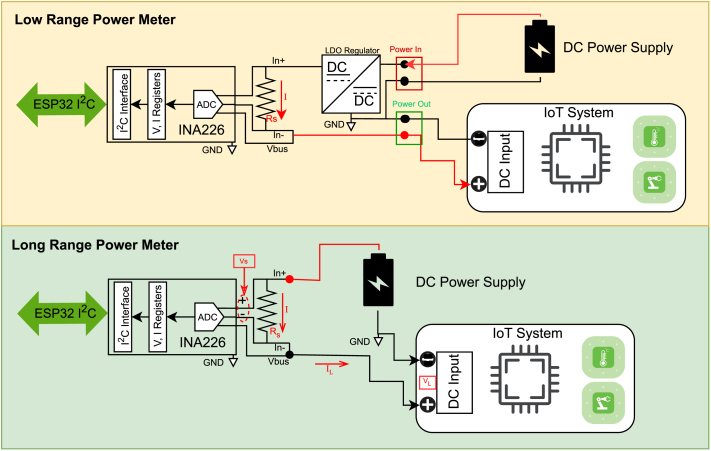
Wiring diagram for connecting a Device Under Test (DUT) and power supply to either the Low-Range or High-Range inputs.

1. **Storage Preparation:** Format a microSD card (max 32 GB) with the FAT32 file system. Create a directory named /Config in the root and place the Config.txt file inside.
2. **Wiring the DUT:** Connect the Device Under Test (DUT) and the power source to the appropriate terminals as shown in Fig. 4. Ensure polarity is correct.
3. **Power Up:** Connect the measurement system via the USB-C port or the auxiliary power terminals. The OLED display will show the boot sequence.

### 6.3 Configuration

System behavior is defined in the Config.txt file. This plain-text file allows users to select the active sensor for trigger mode, define Wi-Fi credentials for NTP time synchronization, and apply calibration factors. Table 5 details the key parameters.

**Table 5:** Key configuration file parameters (Config.txt).

Parameter	Description	Example
WIFI_SSID/PASS	Wi-Fi Credentials for NTP time sync.	MyNetwork
TRIGG_POW_SEN	Active sensor for Burst Mode (1 = Low, 2 = High).	1
LR_CUR_RES	Shunt resistance (Ω) for Low Range.	0.1
LR_CUR_COR	Calibration slope for Low Range Current.	0.95
HR_CUR_COR	Calibration slope for High Range Current.	0.943
RES_FILE_HEADER	Custom CSV header for UART metadata logging.	%Samples,Time

### 6.4 Operating modes

**Fig. 5:**
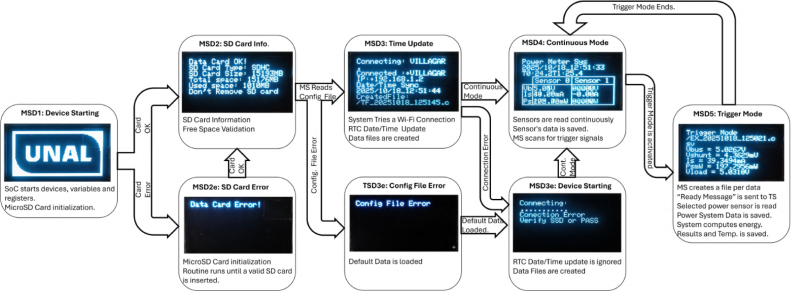
Operational diagram of the measurement system illustrating the transition between initialization, continuous monitoring, and triggered acquisition states.

The system logic flow is summarized in Fig. 5.

#### 6.4.1 Continuous mode (Default)

Upon successful boot, the system automatically enters Continuous Mode.

• **Function:** Real-time visualization of electrical variables on the OLED display. The system logs 1-minute averaged statistics to the SD card.
• **Output File:** Data is appended to CN_YYYYMMDD_hhmmss.csv. The recorded fields are described in Table 6 (Top) Continuous Log file.
• **Usage:** Ideal for long-term profiling or characterizing steady-state loads.

#### 6.4.2 Triggered mode (High-speed)

This mode captures high-resolution transients synchronized with external events.

1. **Activation:** Triggered via a logic-high signal (3.3 V) or UART command.
2. **Output Files:** Two files are generated per event:
  • EX_YYYYMMDD_hhmmss.csv (Burst Raw Data): High-frequency time series (Voltage/Current/Power) for waveform analysis.
  • TF_YYYYMMDD_hhmmss.csv (Burst Metadata): Experiment summary containing total energy, duration, and test metadata (see Table 6).

**Table 6:** Description of data fields stored in the system log files (Continuous, Raw Burst, and Metadata).

File type	Key fields	Description/Significance
Continuous log	Time stamp	YYYYMMDD hhmmss
	MeanVb/MeanP	1-minute averaged Bus Voltage (V) and Power (mW)
	MeanT0/MeanT1	Temperature (°C) for environmental thermal profiling

Burst raw data	Time (μs)	High-resolution timestamp from trigger start
	$V_{bus}$/$I_{s}$/$P_{s}$	Instantaneous Voltage (V), Current (mA), and Power (mW) @ 500 Hz

Burst metadata	**Energy (J)**	**Total energy consumed during the event (Trapezoidal integration)**
	Duration (μs)	Total execution time of the algorithm/event
	Temperature (°C)	Average SoC and/or thermistors temperatures, during the specific event
	Algorithm params	Test context (e.g., $R^{2}$, Dataset size %) received via UART

### 6.5 Electrical sensor parameter adjustment

To minimize systematic errors due to component tolerances, a functional adjustment procedure is recommended using a reference instrument.

**Required Equipment:**

• A stable DC Power Supply (variable 1 V–30 V).
• A Reference Multimeter (e.g., **Fluke 179 True RMS**).
• A resistive load (e.g., 100Ω/10 W for Low Range).

**Adjustment Procedure:**

1. **Reset Configuration:** Open Config.txt and set all correction factors to default values (Slope = 1.0, Offset = 0.0).
2. **Connections:** Connect the power supply and load. Connect the Reference Multimeter to measure the actual voltage ($V_{ref}$) and current ($I_{ref}$).
3. **Sweep & Record:** Vary the input voltage across the operating range. Record the values displayed on the OLED screen ($Val_{measured}$) and the Multimeter ($Val_{ref}$).
4. **Calculation:** Calculate the correction factor (m) using linear regression or the ratio $m=Val_{ref}/Val_{measured}$.
5. **Apply:** Update the Config.txt file with the calculated m value (e.g., LR_VOL_COR). Save and restart.

*Note: Validation results of this procedure are presented in* Section 7*.*

## 7 Validation and characterization

To evaluate the metrological performance and functional capabilities of the proposed hardware, the system underwent a two-stage validation process. First, a static characterization was performed to verify the sensor adjustment parameters derived from manual reference measurements. Second, a dynamic validation was conducted using the “Validation Node” to verify the system’s ability to profile high-speed IoT workloads.

### 7.1 Static characterization and parameter adjustment

A static characterization was performed to determine the linear correction factors required to compensate for component tolerances (e.g., shunt resistor deviations). It is important to note that this process constitutes a **functional parameter adjustment** rather than a strict metrological calibration.

#### 7.1.1 Experimental setup and data acquisition method

To obtain the adjustment parameters and fully characterize the measurement error over the operational range of the INA226 modules, a controlled static data acquisition method was executed. The experimental setup utilized a stable, variable DC power supply (1 V to 30 V) and a Fluke 179 True RMS Multimeter acting as the reference working standard.

To systematically sweep the current and voltage profiles, high-power wirewound rheostats were employed as purely resistive, dynamic loads. For the High-Range channel, a 10 Ω/ 200 W rheostat was selected. This allowed for continuous current sweeps up to 3 A without inducing thermal degradation or resistance drift caused by Joule heating. Conversely, a 100 Ω/ 50 W rheostat was implemented for the Low-Range channel, providing fine-grained control to sweep currents up to 600 mA with high precision.

For each channel, 49 discrete measurement points were systematically recorded. At each interval, the input voltage or resistance was adjusted, and simultaneous readings were captured from both the proposed energy monitoring platform and the reference multimeter. This rheostat-based methodology ensured highly stable loads, minimizing dynamic fluctuations and properly isolating the static metrological performance of the sensors across their entire measurement range.

#### 7.1.2 Reference standard justification

A **Fluke 179 True RMS Multimeter** was selected as the reference instrument (Working Standard).

• **Accuracy vs. Tolerance:** The Fluke 179 offers a DC voltage accuracy of ±(0.09%+1) and current accuracy of ±(1.0%+3). This precision is an order of magnitude superior to the unadjusted tolerance chain of the low-cost INA226 modules and shunt resistors (typically 1%).
• **Suitability:** Consequently, the multimeter provides a valid ground truth for minimizing systematic gain and offset errors in this tier of open-hardware instrumentation.

#### 7.1.3 Adjustment mechanism (Firmware-agnostic and least squares calibration)

A key feature of the proposed hardware is its configuration flexibility. Unlike typical embedded meters that require firmware recompilation to update calibration constants, this system reads the adjustment parameters from the Config.txt file on the microSD card at boot time. To derive these parameters, a least-squares linear regression (y=mx+b) was performed, comparing the uncalibrated raw measurements against the working standard (Fluke 179). This architecture allows researchers to compute and apply the derived slope (m) and offset (b) directly in the field using only a text editor, significantly enhancing maintainability.

#### 7.1.4 Linearity results

To validate the calibration and perform a rigorous error analysis, 49 measurement points were collected manually across the operating range (1.2 V to 30 V; 0 A to 3 A). Table 7 summarizes the regression results. The determination coefficients ($R^{2}>0.9999$) confirm the high linearity of the sensors, validating the use of the linear model. The derived slopes (m) were directly implemented as the correction factors in the configuration file.

**Fig. 6:**
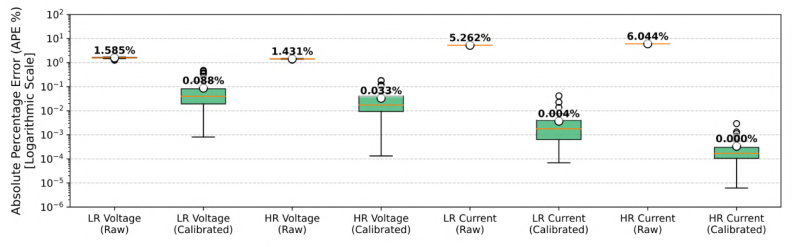
Absolute Percentage Error (APE) distribution for voltage and current channels, comparing raw and calibrated measurements on a logarithmic scale.

To explicitly quantify the accuracy improvements, Fig. 6 presents the Absolute Percentage Error (APE) distribution. The uncalibrated measurements exhibited a Mean Absolute Percentage Error (MAPE) ranging between 5.5% and 7.1% due to component tolerances. By applying the least-squares correction factors, the systematic error was effectively mitigated, reducing the MAPE to negligible levels (<0.08% for all voltage and current channels).

**Table 7:** Parameter adjustment results obtained from linear regression comparison against Fluke 179 reference.

Sensor module	Parameter	Slope (m)/Correction factor	Offset (b)	Linearity ($R^{2}$)
Low-Range (INA226)	Voltage	0.9844	−0.0025 V	>0.9999
	Current	0.9500	＋0.0027 mA	>0.9999

High-Range (INA226)	Voltage	0.9859	−0.0003 V	>0.9999
	Current	0.9430	＋0.0004 mA	>0.9999

*Note*: The Slope (m) is entered into Config.txt as LR_VOL_COR, LR_CUR_COR, etc. Offsets (b) were found to be negligible (<0.05% of full scale) but can also be configured if required.

**Fig. 7:**
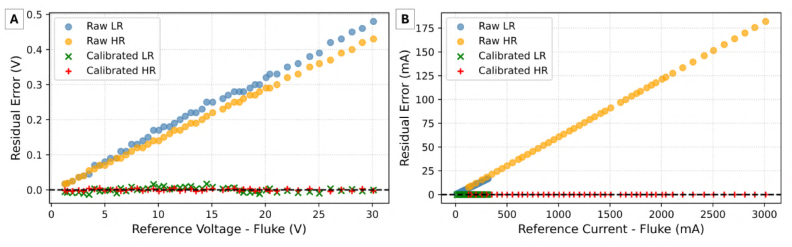
Residual error analysis for voltage and current channels, demonstrating the mitigation of systematic deviation following least-squares calibration. (A) Voltage Error, (B) Current Error.

Furthermore, the residual scatter analysis shown in Fig. 7 demonstrates that the raw systematic deviation, which is proportional to the magnitude, is completely flattened around the zero axis across the entire dynamic range. This confirms that post-calibration, the measurement uncertainty is bounded almost entirely by the limits of the reference instrument rather than the sensing hardware.

#### 7.1.5 Uncertainty analysis, quantization, and thermal stability

To provide a rigorous estimation of the system’s accuracy, an uncertainty budget was formulated encompassing the reference instrument, sensor quantization, thermal drift effects, and numerical integration limits.

**Reference Uncertainty Budget:** The adjustment parameters were derived using a Fluke 179 True RMS Multimeter as the working standard. According to the manufacturer’s specifications [13], the absolute uncertainty ($U_{ref}$) for DC measurements is defined as $U_{ref}=\pm \left(\text{\% reading}+\text{counts}\right)$. For the ranges used in this characterization:

• Voltage (60 V Range): Accuracy of ±(0.09%+1). At 5 V, $U_{V}\approx \pm 5.5\;\text{mV}$.
• Current (600 mA/10 A Ranges): Accuracy of ±(1.0%+3). At 500 mA, $U_{I}\approx \pm 5.3\;\text{mA}$.

Since the correction factors are derived directly from this reference, the measurement system’s baseline uncertainty is bounded by $U_{ref}$ plus the quantization error of the ADC.

**Sensor Quantization (INA226):** The INA226 incorporates a 16-bit delta-sigma ADC [14]. The bus voltage register possesses a fixed Least Significant Bit (LSB) of 1.25mV, and the shunt voltage register provides an LSB of 2.5μV. To optimize the signal-to-noise ratio while maintaining a high sampling rate ($f_{s}\approx 500\;\text{Hz}$), the ADC was configured with a conversion time of 140μs and an averaging mode of 4 samples. This hardware-level averaging suppresses transient thermal noise without degrading the temporal resolution required for Edge-AI profiling.

The baseline current quantization error ($I_{LSB}$) is strictly a function of the selected shunt resistor ($R_{shunt}$), defined as $I_{LSB}=\frac{2.5\;\mu \text{V}}{R_{shunt}}$. Consequently, the theoretical current quantization limits are 25μA for the Low-Range Channel ($R_{shunt}=0.1\;\Omega$) and 250μA for the High-Range Channel ($R_{shunt}=0.01\;\Omega$).

**Thermal Drift (Joule Heating):** A critical source of dynamic error in resistive current sensing is the change in shunt resistance due to self-heating, governed by the Joule effect ($P=I^{2}R$ with P in [W], I in [A] and R in [Ω]) and the Temperature Coefficient of Resistance (TCR) $\alpha_{TCR}$ [15]. The resistance deviation ΔR[Ω] due to temperature rise ΔT[°C] is given by $R\left(T\right)=R_{0}\left[1+\alpha_{TCR}\left(T-T_{0}\right)\right]$ in [Ω], where $\alpha_{TCR}$ in [ppm/°C] (typically ≈50−100ppm/°C for metal film resistors as used in the assembly) and $R_{0}\;\left[\Omega \right]$ is the nominal value of shunt resistor at $T_{0}=25\;\left[{}^{\circ}\text{C}\right]$.

• Low-Range Scenario: With a maximum current of 600 mA through a 0.1Ω resistor, power dissipation is negligible (P≈36mW), resulting in minimal thermal drift.
• High-Range Scenario: At the continuous limit of 3 A through the 0.01Ω shunt, power dissipation is 0.09 W. During 8 A bursts, dissipation rises to 0.64 W. While the resistor is rated for this power, the resulting temperature rise temporarily increases $R_{shunt}$, introducing a dynamic gain error estimated at <0.1% for short bursts (<10s).

This confirms that for the intended IoT workloads (pulsed loads), thermal drift remains below the reference uncertainty threshold.

**Propagation of Uncertainty in the Developed Measurement System:** The total energy (E) in [J] is computed via numerical integration of the instantaneous power (P=V⋅I P in [W], V in [V] and I in [A]) using the trapezoidal rule over discrete sampling intervals (Δt in [s]). To determine the measurement confidence of the developed hardware, the uncertainty must be propagated from the independent voltage and current measurements ($U_{V}$ in [V] and $U_{I}$ in [A]) to the final energy value.

Since the voltage and current are acquired as statistically independent variables by the INA226 ADCs, the combined standard uncertainty for the instantaneous power ($U_{P}$ in [W]) is derived using the first-order Taylor series expansion (propagation of error):

(1) $$U_{P}=P\sqrt{{\left(\frac{U_{V}}{V}\right)}^{2}+{\left(\frac{U_{I}}{I}\right)}^{2}}=\sqrt{{\left(I\cdot U_{V}\right)}^{2}+{\left(V\cdot U_{I}\right)}^{2}}\;\left[W\right]$$

Assuming the uncertainty of the hardware timer (Δt) is negligible due to the precision of the ESP32-S3 internal clock, the uncertainty propagates directly into the discrete energy integral:

(2) $$E\approx \overset{N-1}{\underset{k=1}{\sum }}\frac{P_{k}+P_{k+1}}{2}\Delta t\;\left[\text{J}\right]$$

The propagation of Energy error $U_{E,total}=U_{E,systematic}+U_{E,random}\;\left[\text{J}\right]$ depends on the nature of the uncertainty:

1. **Random Error (Noise):** If the residual uncertainties $U_{V}$ and $U_{I}$ behave as independent, zero-mean high-frequency noise across samples, the error adds in quadrature. The total absolute uncertainty of the calculated energy ($U_{E,random}$) becomes:
  (3) $$U_{E,random}\approx \Delta t\sqrt{\overset{N}{\underset{k=1}{\sum }}U_{P_{k}}^{2}}\;\left[\text{J}\right]$$
  Because it scales with the square root of the number of samples ($\sqrt{N}$), high-frequency random noise is heavily attenuated by the integration process over time.
2. **Systematic Error (Worst-Case Bound):** For calibration offsets or static non-linearities that persist across the entire measurement window, the errors add linearly. In this absolute worst-case scenario, the relative uncertainty of the total energy is bounded by the relative uncertainty of the average power, Where $\overset{¯}{V}$ is the average voltage in [V] and $\overset{¯}{I}$ is the average current in [A] such that:
  (4) $$\frac{U_{E,systematic}}{E}\approx \sqrt{{\left(\frac{U_{V}}{\overset{¯}{V}}\right)}^{2}+{\left(\frac{U_{I}}{\overset{¯}{I}}\right)}^{2}}$$

This mathematical formulation demonstrates that numerical integration inherently filters out zero-mean quantization noise from the INA226, meaning the final energy estimation error is fundamentally bottlenecked only by the residual systematic uncertainties ($U_{V}$ and $U_{I}$) remaining after the least-squares adjustment.

**Overall Uncertainty Budget:** Table 8 summarizes the individual uncertainty contributors. The raw shunt tolerance (typically 1%) introduces a systematic gain error, which is effectively nullified via the least-squares calibration.

To explicitly link this theoretical uncertainty budget to practical Edge-AI workloads, statistical error analysis — including standard deviation and relative percentage uncertainty across multiple experimental iterations — has been visually incorporated into the dynamic validation results (see Section 7.3). This comprehensive error analysis confirms that the observed energy variations during the algorithm execution are statistically robust and strictly bounded by the characterized hardware uncertainty.

**Table 8:** Comprehensive uncertainty budget for the energy monitoring system.

Uncertainty source	Parameter/Method	Low-range channel	High-range channel
Reference instrument	Voltage accuracy	±(0.09%+1count)	±(0.09%+1count)
(Fluke 179)	Current accuracy	±(1.0%+3counts)	±(1.0%+3counts)

Sensor quantization	Voltage resolution	1.25mV LSB	1.25mV LSB
(INA226)	Current resolution	$25\;\mu \text{A LSB}\left(R_{shunt}=0.1\;\Omega \right)$	$250\;\mu \text{A LSB}\left(R_{shunt}=0.01\;\Omega \right)$

Hardware components	Shunt tolerance	1% (Mitigated via cal.)	1% (Mitigated via cal.)

Computational method	Energy propagated error ($U_{E}/E$)	$\approx \sqrt{{\left(\frac{U_{V}}{\overset{¯}{V}}\right)}^{2}+{\left(\frac{U_{I}}{\overset{¯}{I}}\right)}^{2}}$	$\approx \sqrt{{\left(\frac{U_{V}}{\overset{¯}{V}}\right)}^{2}+{\left(\frac{U_{I}}{\overset{¯}{I}}\right)}^{2}}$

### 7.2 Thermal profiling capabilities (Continuous mode)

To validate the system’s sensitivity to environmental variables, a 12-hour continuous logging test was conducted. The Measurement System recorded ambient temperature (via NTC probes) and the DUT’s power consumption simultaneously.

**Fig. 8:**
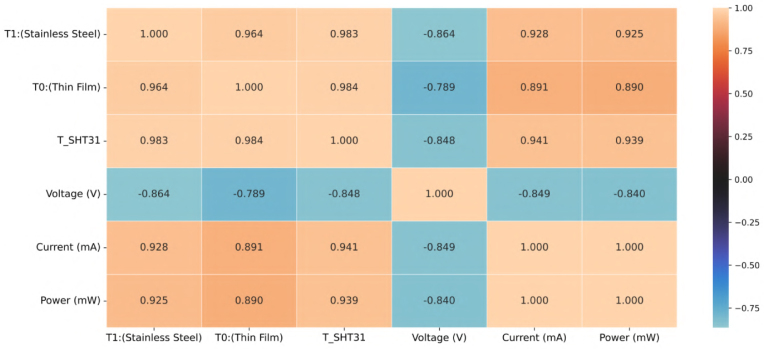
Correlation heatmap between temperature sensors (SHT31, NTCs) and electrical variables. The strong correlation (R>0.89) between Temperature and Power confirms the system’s ability to detect thermally-induced leakage currents.

**Results:** The data revealed a strong positive correlation between ambient temperature and the static power consumption of the ESP32-S3 DUT. As shown in the correlation matrix (Fig. 8), the measured current correlates with temperature (R≈0.90).

• **Physical Interpretation:** This behavior is consistent with semiconductor physics, where leakage currents in CMOS circuits rise exponentially with temperature ($P_{static}\propto e^{T}$) [16].
• **Significance:** The ability of the proposed hardware to capture this subtle thermal-power dependency validates its resolution for fine-grained environmental profiling.

### 7.3 Dynamic validation: Edge-ML case study

Finally, the system was validated in a realistic Edge-AI scenario: profiling the energy cost of training a Linear Regression model on-chip. This demonstrates the utility of the “Triggered Mode” for optimizing embedded software.

To validate the system’s ability to profile high-speed transient loads, a controlled experiment was conducted using the **Validation Node** (detailed in Appendix).

#### 7.3.1 Experimental setup

Instead of using a static resistor, the Validation Node (ESP32-S3) was configured to act as a dynamic load by executing a Linear Regression algorithm trained via Ordinary Least Squares to find the optimal coefficients (slope α and intercept β) that minimize the Mean Squared Error (MSE). While conceptually simple, this workload involves iterative floating-point matrix operations typical of the core kernels found in more complex Machine Learning training tasks

This setup serves two purposes:

1. **Load Generation:** It produces realistic current spikes typical of Edge-AI processing.
2. **Synchronization:** It generates a hardware trigger signal exactly 70 ms before the computation starts, allowing the Measurement System to verify its “Triggered Mode” latency and capture window.

#### 7.3.2 Workload characterization

**Fig. 9:**
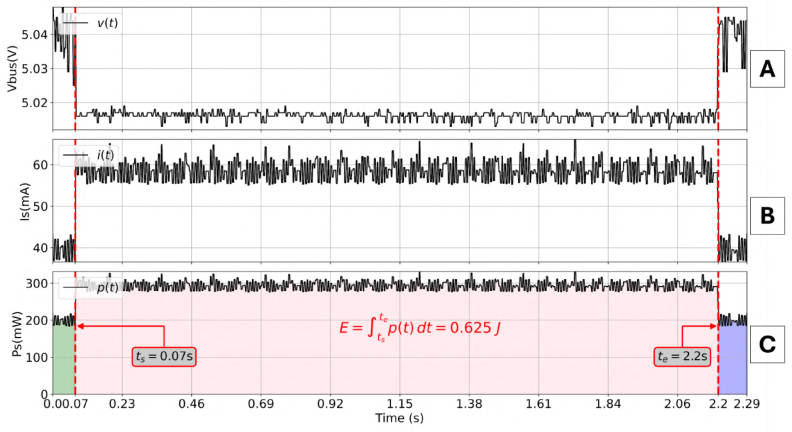
Dynamic profile of a Linear Regression training task (100% dataset). The Measurement System captures the transitions between Idle and Active states with high fidelity ($f_{s}\approx 500\;\mathrm{Hz}$). Measured Electric Variables: (A) Voltage, (B) Current, (C) Power.

The Validation Node executed the training algorithm on the “Red Wine Quality” dataset [17], varying the training set size from 1% to 100%. Fig. 9 shows the electrical signature of a full-dataset training run.

• **Temporal Resolution:** The system successfully captured the 2.13 s execution window. The logic trigger provided precise synchronization, isolating the algorithm energy ($E_{algo}$) from the baseline.
• **Current Profile:** The average current rose from ≈39 mA (Idle) to ≈57 mA (Active), a 46% increase attributable to CPU and memory activity. Voltage remained stable (5.03V±0.4%), confirming the robustness of the Low-Range regulator.

#### 7.3.3 Energy vs. Accuracy trade-off

A key application of this hardware is finding the optimal operating point for battery-powered devices. By correlating the energy measurements from the hardware with the model’s performance metrics ($R^{2}$, MSE) recorded in the metadata, we identified a non-linear trade-off.

**Fig. 10:**
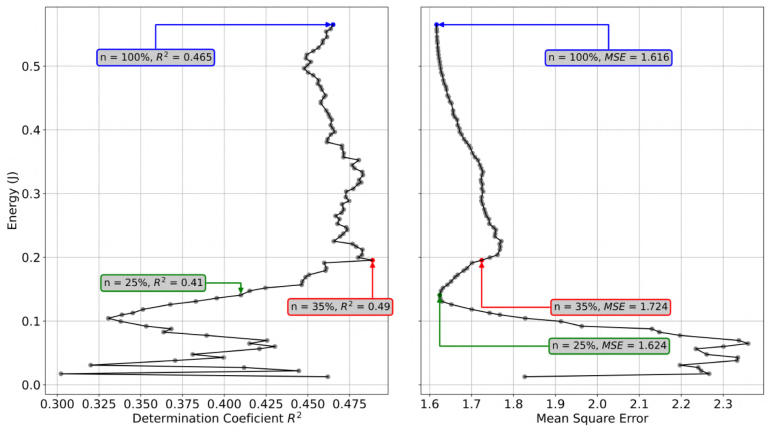
Energy vs. Performance trade-off. The “Pareto front” shows that increasing energy expenditure (y-axis) beyond 0.2 J yields diminishing returns in model accuracy ($R^{2}$).

As illustrated in Fig. 10:

1. **Energy Cost:** Energy consumption scales linearly with dataset size (0.15 J at 25% → 0.62 J at 100%).
2. **Model Performance:** The determination coefficient ($R^{2}$) plateaus quickly. Using just 35% of the data achieves an $R^{2}\approx 0.49$, which is superior to the result obtained with 100% data ($R^{2}\approx 0.47$) due to overfitting or data redundancy.
3. **Conclusion:** The hardware enabled the identification of an optimal “green” operating point (35% data), saving **65% of energy** with no loss in predictive accuracy.

#### 7.3.4 Energy calculation stability and error estimation

To explicitly evaluate the stability and dynamic calculation error of the energy measurement system, an independent experimental phase was conducted. During this phase, the linear regression training algorithm was executed while systematically varying the dataset proportion from 10% to 100%. To acquire a statistically significant sample and isolate measurement variance, the execution for each discrete subset was repeated five times.

**Fig. 11:**
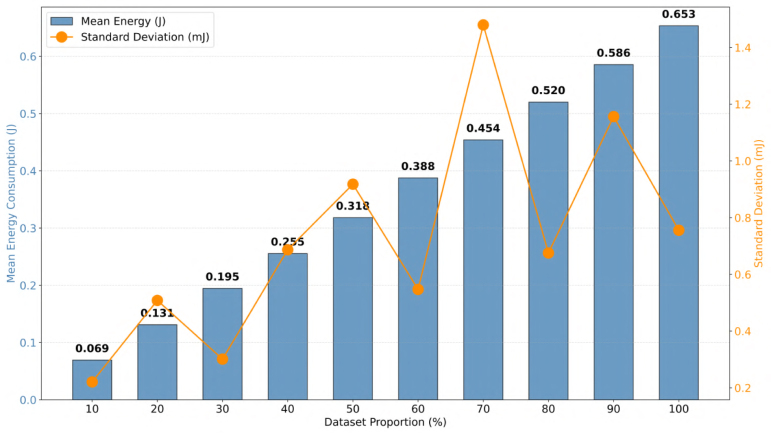
Dual-axis energy profiling: mean consumption (blue bars, primary axis) and sub-millijoule standard deviation (orange line, secondary axis) across varying dataset proportions (10%–100%).

This repetitive sampling strategy evaluates the variance inherent to the hardware’s numerical integration (trapezoidal rule) under real Edge-AI workloads. Because the dynamic measurement error is significantly smaller than the total energy magnitude, Fig. 11 employs a dual-axis representation. The primary axis displays the linear progression of the mean energy consumption, while the secondary axis explicitly plots the standard deviation (in milliJoules).

As depicted, the absolute variance remains remarkably bounded across all operational states (fluctuating between ≈0.2mJ and 1.4mJ). Quantitatively, the measurement system demonstrated an average relative percentage error (Coefficient of Variation) of only 0.23% across all executed profiles, peaking at a maximum of just 0.38%.

These tightly constrained, sub-percent error margins empirically demonstrate that the real-time energy calculation is highly stable. It confirms that the systematic measurement error propagates minimally across continuous training cycles, validating the platform’s reliability for granular energy profiling in resource-constrained IoT environments.

### 7.4 Comparison with state-of-the-art solutions

To assess the practical value of the proposed hardware, Table 9 compares its specifications against industry-standard profilers (Nordic), popular consumer USB testers (RD/Fnirsi), and basic DIY implementations.

While commercial USB testers like the *Fnirsi FNB58* or *Ruideng UM25C* offer standalone operation and integrated displays similar to our proposal, they lack the **synchronization capabilities** required for scientific research. They cannot trigger acquisition based on code execution states, nor can they log algorithmic metadata (e.g., model accuracy) alongside the power data. The proposed system bridges this gap, offering the autonomy of a USB tester with the triggering features of a professional profiler like the PPK2, at a fraction of the cost.

**Table 9:** Comparison of the Proposed system vs. Commercial and DIY alternatives.

Feature	Proposed system	Nordic PPK II	USB testers (e.g., FNB58, UM25C)	Basic DIY (INA219)
Primary use case	**Edge-AI/IoT profiling**	Professional low-power Dev	Charger/Battery diagnostics	Hobbyist logging
Approx. cost	<$65**USD**	≈$500 USD	$30−$50 USD	<$15 USD
Standalone mode	**Yes (OLED + SD)**	No (USB tethered)	Yes (OLED)	Depends on MCU
Sampling rate	≈500Hz	100 kHz	10−100Hz (Log)	10−100Hz
Sync/Trigger	**Hardware GPIO + UART**	Digital input	None	None
Data context	**Automated metadata (TF files)**	Manual annotation	None (Raw V/I only)	Raw CSV
Voltage range	0−30 V (Dual range)	0.8−5 V	0−24 V	0−26 V

### 7.5 System limitations

Despite its utility for general IoT profiling, users must be aware of the following intrinsic limitations:

• **Bandwidth vs. Transients:** With a sampling rate of ≈500Hz, the system effectively captures the energy envelope of software tasks (duration >10ms). However, it behaves as a low-pass filter for fast hardware transients. It cannot resolve sub-millisecond inrush currents (e.g., Wi-Fi beacon spikes) which require sampling rates in the kHz/MHz range.
• **Low-Current Resolution:** The use of an onboard DC-DC buck converter introduces switching noise. While the dual-shunt design improves dynamic range, the noise floor limits the effective resolution in the deep-sleep range (<10μA). For strict nano-power characterization, linear regulation or battery-only operation would be required.

### 7.6 Failure modes and reliability

Testing revealed two primary failure modes that users should mitigate:

• **SD Card Latency:** High-speed logging is sensitive to SD card write latency. Using low-quality cards (Class 4 or lower) can cause buffer overflows, resulting in dropped samples during long acquisitions.
• **Thermal Derating:** As noted in Section 6, the PCB trace width is the limiting factor for high currents. Prolonged operation at >5 A without active cooling can lead to trace delamination or solder joint fatigue due to thermal cycling.

### 7.7 Conclusions and future work

This work presented the design and validation of an open-source, dual-channel energy monitoring system explicitly tailored for Edge-AI applications. By eliminating the dependence on expensive commercial multimeters or full-scale single-board computers, this tool effectively bridges the gap between hardware power profiling and software execution at the edge.

Quantitatively, the measurement architecture demonstrated exceptional accuracy following the implementation of a least-squares calibration method, reducing the Mean Absolute Percentage Error (MAPE) to negligible levels (<0.08%) across both voltage and current channels. During the dynamic profiling of a linear regression algorithm, the platform successfully captured the linear scalability of energy consumption, explicitly quantifying that reducing the dataset proportion to 50% yields a proportional energy saving of 51.6%. Furthermore, the stability of the real-time energy calculation was empirically validated, exhibiting an average relative percentage error (Coefficient of Variation) of merely 0.23%. This tightly bounded variance directly corroborates the theoretical uncertainty propagation model, confirming that the numerical integration effectively attenuates high-frequency quantization noise without degrading the overall measurement confidence.

Beyond algorithmic profiling, the dual-channel architecture significantly expands the platform’s applicability. It enables the simultaneous power monitoring of diverse physical subsystems, such as photovoltaic energy harvesting circuits, wireless communication modules, or active cooling mechanisms. Furthermore, provided the Device Under Test (DUT) supports reprogramming and standard communication protocols, the monitoring platform facilitates the full automation of the data capture process. This capability allows researchers to autonomously generate extensive power-profiling datasets, significantly accelerating the optimization and benchmarking of Edge Machine Learning models on IoT devices. The integration of dual temperature sensors also allows researchers to perform comprehensive energy profiling that directly correlates power consumption with ambient environmental conditions and the specific thermal dynamics of the installation.

Despite its high precision and accessibility, this study acknowledges specific architectural and hardware limitations. While utilizing commercial off-the-shelf (COTS) breakout modules facilitated low-cost and rapid assembly for researchers, it inherently results in a suboptimal physical footprint and a higher baseline power consumption. Additionally, the system’s maximum sampling frequency (≈500Hz) prevents the capture of ultra-fast, sub-millisecond transient current spikes.

Future hardware iterations will address these constraints by migrating from a modular approach to a fully integrated custom Printed Circuit Board (PCB). Consolidating the processing unit, storage, and sensors onto a single substrate will significantly miniaturize the system. Concurrently, implementing rigorous low-power firmware strategies will be prioritized to minimize the monitoring platform’s own energy footprint, ensuring it exerts negligible influence on the measured environment.

Finally, future development will also leverage the ESP32-S3’s native Wi-Fi and Bluetooth capabilities. Upgrading the firmware to support real-time wireless data transmission will transition the platform from local SD-card storage to seamless cloud integration, providing a scalable, connected foundation for energy-aware artificial intelligence across IoT network infrastructures.

## CRediT authorship contribution statement

**Alvaro A. Villa-Garzón:** Writing – original draft, Visualization, Validation, Methodology, Investigation, Formal analysis, Data curation, Conceptualization. **Jhon W. Branch-Bedoya:** Writing – review & editing, Resources, Project administration. **Fernan A. Villa-Garzón:** Writing – review & editing, Validation, Methodology, Formal analysis.

## Ethics statements

“The work described in this article did not involve human subjects or animal experiments”.

## Declaration of competing interest

The authors declare that they have no known competing financial interests or personal relationships that could have appeared to influence the work reported in this paper.

## Figures and Tables

**Fig. 12:**
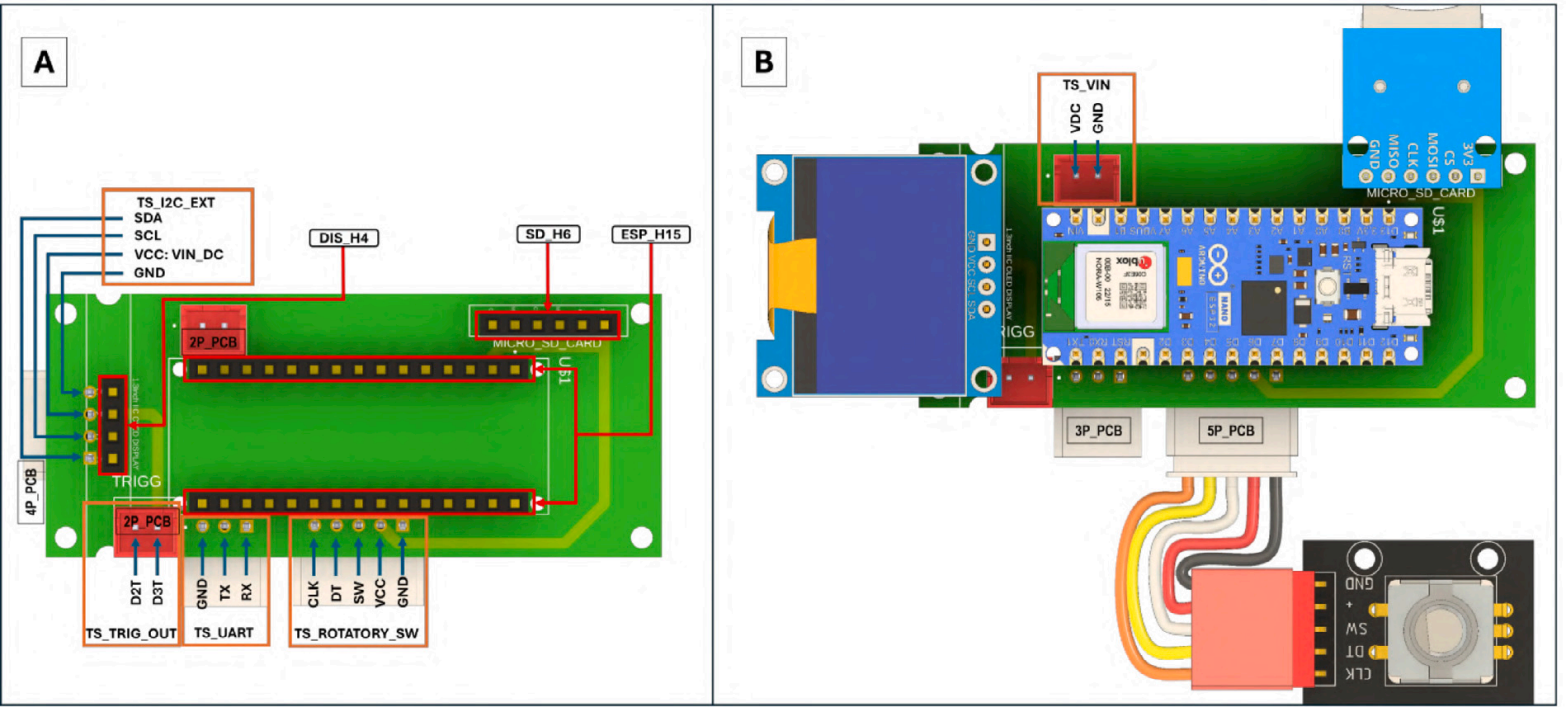
Hardware implementation of the Validation Node. (A) PCB Layout exposing UART and Trigger terminals. (B) Assembled unit connecting the Rotary Encoder and OLED display.

**Fig. 13:**
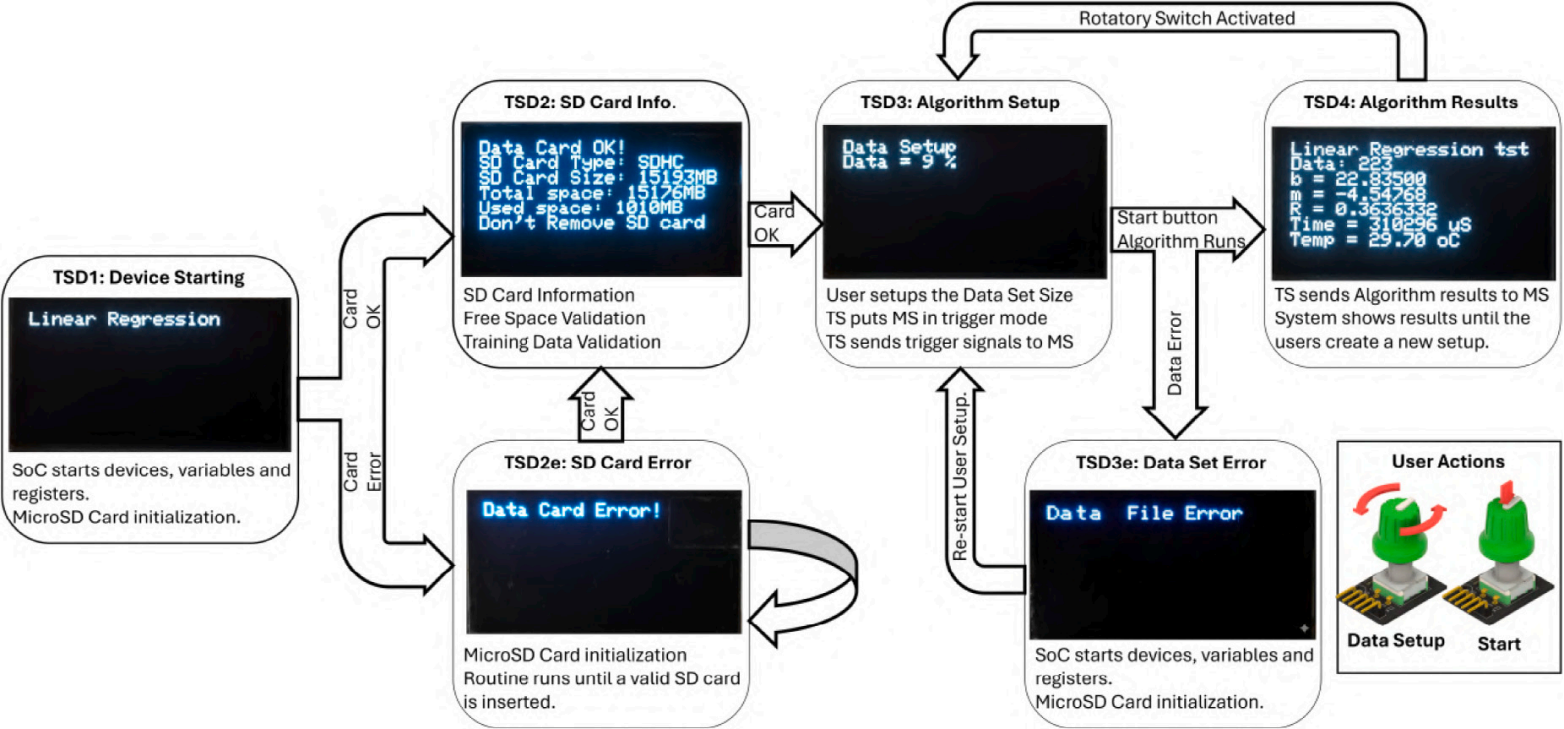
Operational workflow of the validation node. The diagram illustrates the user interaction via the rotary switch to select the workload intensity and the resulting visual feedback.

## Appendix Validation node (Test system) design

To validate the measurement platform under realistic conditions, a standalone “Validation Node” was developed. This device acts as a programmable dynamic load, capable of executing a computational workload (Linear Regression) with variable intensity while generating hardware synchronization signals.

### A.1 Hardware implementation

The node is built around an **Arduino Nano ESP32 (ESP32-S3)** and operates independently from the main measurement system. As shown in Fig. 12, the design integrates:

• **User Interface:** A 1.3-inch OLED display (SH1106) and a rotary encoder (KY-040) allow the user to configure the experiment parameters (e.g., training dataset size) without a PC connection.
• **Synchronization Interface:** Dedicated GPIO pins output a 3.3 V logic signal to trigger the Measurement System immediately before the algorithm begins and de-assert it upon completion.
• **Power:** An integrated 18650 Li-Ion battery holder with a 5 V boost converter ensures autonomous operation, isolating the ground loops during field testing.

### A.2 Firmware and operation

The firmware implements a standard Gradient Descent Linear Regression algorithm using the “Red Wine Quality” dataset stored on a microSD card. The operational flow (Fig. 13) consists of three stages:

1. **Configuration:** The user rotates the encoder to select the percentage of the dataset to be processed (1% to 100%).
2. **Execution & Triggering:** Upon confirmation, the system pulls the TRIG_OUT pin HIGH, executes the training loop, and pulls the pin LOW.
3. **Reporting:** Performance metrics ($R^{2}$, execution time, coefficients) are displayed on the OLED and transmitted via UART to the Measurement System for logging.

All design files (Gerber, BOM, and Firmware) for this node are available in the repository to facilitate the replication of the experimental setup.
